# Expanding the Riboglow-FLIM Toolbox with Different Fluorescence Lifetime-Producing RNA Tags

**DOI:** 10.1021/acs.biochem.4c00567

**Published:** 2025-05-15

**Authors:** Zachary Stickelman, Nadia Sarfraz, Morgan K. Rice, Ben J. Lambeck, Sonja Milkovich, Esther Braselmann

**Affiliations:** Department of Chemistry, 8368 Georgetown University, Washington, District of Columbia 20057, United States

## Abstract

RNAs are essential elements of biology with subcellular localizations critical for function. Genetically tagging fluorescent RNA reporters to an RNA of interest allows for investigating RNA spatiotemporal dynamics. We previously developed a fluorescence lifetime imaging microscopy (FLIM)-based RNA-tagging platform, Riboglow-FLIM. Here, a genetically encoded RNA tag binds a fluorescent probe, causing an increase in both fluorescence intensity and fluorescence lifetime. Importantly, the Riboglow platform is derived from a bacterial riboswitch RNA family and different riboswitch sequences from nature may build the basis for multiplexing capabilities. We previously observed fluorescence lifetime differences for two RNA tags *in vitro* and in live mammalian cells as a proof-of-concept demonstration. As an in-depth expansion for multiplexing capabilities, here we evaluate the performance of different RNA sequences *in vitro* for systematically expanding the RNA tag sequence space of Riboglow-FLIM. We use two methods of varying the genetic tag to evaluate multiplexing capabilities, a literature-guided and a rational design approach. The literature-guided approach includes riboswitch sequences with both indirect and direct evidence of probe binding. For this, a phylogenetic tree of riboswitch-derived tags from indirect binding results was constructed, and RNA members from different branches were characterized. We also designed RNA mutations rationally based on insights from established Riboglow RNA tags. Together, nine different RNA tags yielded a wide range of fluorescence lifetimes for the Riboglow-FLIM platform, building the foundation to tag and track several different RNAs simultaneously. These findings will serve as the basis for achieving multiplexed RNA imaging in live cells using a fluorescence lifetime sensor.

## Graphical abstract

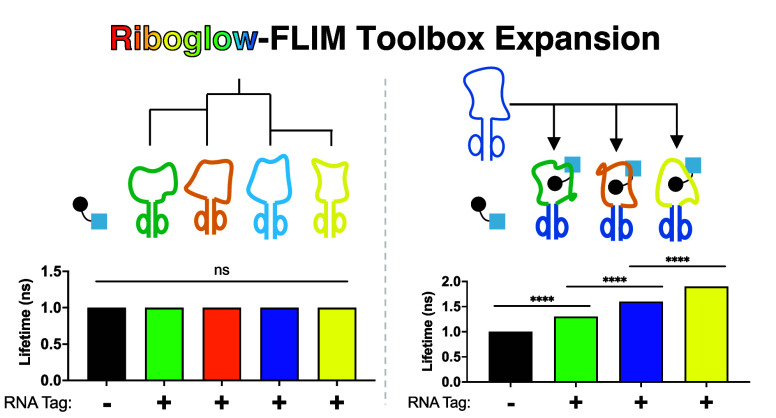

## Introduction

Since the 1950s, the Central Dogma has described the flow of biological information from the storage of genetics as DNA to the expression of genetics as proteins, with messenger RNA (mRNA) being the facilitator.^1^ Since then, diverse additional functionalities of RNAs have been recognized: RNAs can act as gene regulators^2^ and can even have catalytic activity similar to proteins.^3^ Noncoding RNAs (ncRNAs) have functions beyond roles as protein-coding mRNAs and modulate functions beyond protein synthesis.^4^ Although many ncRNAs are being discovered and characterized, the functions of a large proportion are yet unknown.^5^

RNA localization within living cells and organisms is critically tied to their functions^6^ and changes in response to genetic^7^ or environmental^8^ perturbations. Therefore, it is important to investigate and visualize RNA localization, especially to study the functions and spatiotemporal dynamics of RNAs. Several techniques were developed to visualize RNAs in live cells. There are four main groups of fluorescent RNA tools: protein-based, fluorophore-aptamer, dye-quencher, and hybridization.^9^ Protein-based RNA fluorescent sensors utilize natural protein-RNA interactions. An RNA of interest is tagged with a stem-loop RNA (typically derived from bacteriophages) that can bind an RNA-binding protein genetically fused to a fluorescent protein, such as in the MS2 system.^10^ Fluorophore-aptamers are engineered dye-binding aptamers and these RNA aptamers include the Spinach,^11^ Broccoli,^12^ and Mango^13^ tags, as well as the RhoBAST:SpyRho^14^ and Okra505 ^15^ systems. Here, the RNA tag binds a small molecule fluorescent dye for visualization. Dye-quencher systems involve the use of a fluorophore as well as a quencher, causing either a fluorescence increase or decrease upon binding to the RNA, such as our Riboglow system.^16^ Lastly, the hybridization technique (molecular beacons) are oligonucleotide probes with stem-loops that are modified at the termini to contain a fluorophore and quencher that hybridizes to an RNA of interest, separating the fluorophore from the quencher to increase fluorescence.^17^ Because the need to investigate subcellular RNA localizations has been increasingly recognized for understanding their function, several technologies continue to be developed to visualize RNAs in live cells.

While several systems for RNA visualization tools were presented, current approaches limit multiplexing capabilities, or the ability to visualize more than one RNA of interest simultaneously in the same cell. Multiplexed visualization of several RNAs simultaneously is needed as RNAs interact with other RNAs, leading to functions that are tightly regulated and in some cases linked to disease.^5,18^ An example of protein-based RNA visualization for labeling multiple RNAs of interest simultaneously is the MS2 and PP7 systems, where two different RNA stem-loops bind specific RNA-binding protein partners fused to two different fluorescent proteins. This multiplexing proof-of-concept system was demonstrated for the ability to tag mRNAs encoded on two different alleles in yeast and Drosophila embryos.^19–21^ However, the excitation and emission spectra of fluorescent proteins may overlap, limiting the ability to separate tagged RNAs spectrally. One approach of a fluorophore-aptamer platform that may allow the labeling of multiple RNAs of interest involves using orthogonal fluorescent light-up aptamers in live cells, but this approach is similarly limited to the spectra of the dyes used.^22^ Combinations of fluorophore-aptamers that were attempted for multiplexing are Mango/Spinach,^23^ Broccoli/DNB,^24^ and Broccoli/Squash.^25^ However, cross-reactivity between the ligands and RNAs was observed,^23–25^ preventing robust multiplexing in live cells. While current efforts to color-multiplex RNA fluorescent tags are underway, here we explore an alternative and complementary approach to achieve orthogonal visualization of multiple RNAs of interest simultaneously using our Riboglow-FLIM platform.

We previously developed Riboglow-FLIM (Figure 1a), an RNA sensor where a genetically encoded RNA tag binds a small fluorescence probe with nM affinity.^16,26^ The probe consists of cobalamin (Cbl) covalently linked to a fluorophore (i.e., Cy5). Cbl is a fluorescence quencher of the attached fluorophore and fluorescence intensity^16^ and fluorescence lifetime^26^ increase upon RNA tag binding to the Cbl moiety of the probe. Cbl comes in various forms with different β-axial groups, such as cyano- (CN), aqua-, adenosyl-, and methyl-.^27^ All probes for the Riboglow system use the CN-β axial ligand. We previously demonstrated in a proof-of-concept application that changing the RNA sequence induces different probe fluorescence intensities^16^ and lifetimes,^26^ allowing us to visualize two different RNAs simultaneously by monitoring the fluorescence lifetime.^26^ Riboglow was initially characterized with RNA tag variants “A” and “D” (∼100 nucleotide long sequences derived from nature) using fluorescence intensity while using several fluorescent probes.^16^ Each RNA variant exhibits nM affinity to binding Cbl and differentiable fold fluorescence turn-on *in vitro* for most probes,^16^ indicating that the fluorescence readout is sensitive to RNA sequence variants. Fluorescence intensity is highly concentration-dependent, making it difficult to control, especially in live cells where multiplexing is desired.^16^ Fluorescence intensity is directly proportional to the amount of fluorescent material. Therefore, if the amount of fluorescent probe and/or produced sensor RNA in each live cell is different, the fluorescence intensity of each cell will be different, resulting in a broad spread of fluorescence intensity read-out values. This makes multiplexing in cells difficult, where different fluorescence intensity readouts upon binding different RNAs are expected. To overcome the issue of concentration dependence, we explored fluorescent lifetime.^26^ Fluorescence lifetimes of the Riboglow probe upon binding two RNA variants (A- and D-tag) are differentiable *in vitro*.^26^ We previously tagged two model RNAs of interest with the RNA A- and D-tag for simultaneous visualization within live mammalian cells using the probe Cbl-4xGly-ATTO590.^26^ However, to achieve further imaging depths,^28^ especially as we move forward into multicellular organisms such as Zebrafish,^29^ the far red-spectral Riboglow probe Cbl-Cy5 may be advantageous.

**Figure 1:**
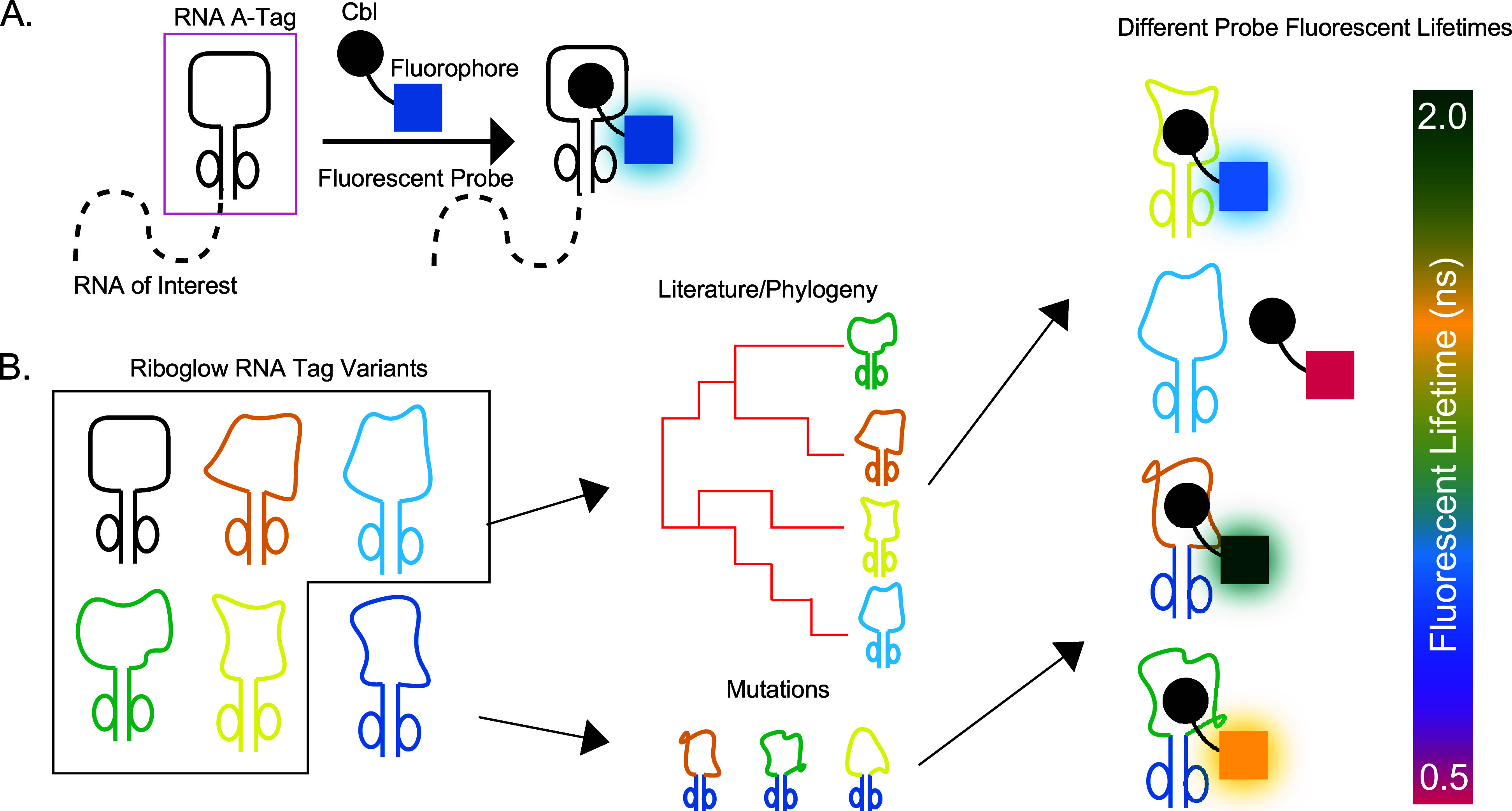
Riboglow platform and workflow. (A) The Riboglow platform consists of a riboswitch-derived RNA tag (pink box) that may be genetically fused to an RNA of interest. The fluorescent probe (i.e., Cbl-Cy5) contains the natural ligand to the riboswitch, Cobalamin (Cbl), coupled to a synthetic fluorophore. Upon RNA/Cbl binding, fluorescence intensity and lifetime of the fluorophore increase. (B) Workflow for expanding Riboglow through varying the RNA tag sequence. All available literature and predicted sequences are cataloged phylogenetically. Other variants are generated through rational mutations. The goal is to generate a series of RNA tags that differ in their fluorescence lifetime upon probe binding. The different colored fluorophore cartoons indicate different fluorescence lifetimes of the same fluorophore molecule.

Here, we explore the RNA tag sequence space systematically to expand the multiplexing capabilities of Riboglow-FLIM. We characterize Cbl-Cy5 probe binding to RNAs obtained through literature-guided and rational mutation-based approaches and evaluate FLIM *in vitro*, leading us to begin understanding principles of RNA-probe binding as it may relate to fluorescence lifetime changes, and we identified nine RNA tag variants with different fluorescence lifetime signatures as candidates for the foundation for multiplexed RNA imaging in live cells.

## Materials and Methods

### RNA Sequence Variants

We selected RNA sequence variant candidates of the Riboglow-FLIM platform by two approaches (SI Table 1). One, a literature-guided approach in which sequences showing either indirect or direct binding to Cbl was compiled. Riboswitch sequences that indirectly indicated binding to Cbl via a Green Fluorescent Protein (GFP) fold repression assay^30^ were used to construct a phylogenic tree. Sequences with direct evidence of Cbl binding were selected that included previously investigated Riboglow tags, as well as other binding assays.^16,26,30–32^ Two, rational mutations of established Riboglow RNA sequences^16,26^ were designed.

### Phylogenetic Tree Preparation

The sequences used for the phylogenetic tree preparation were previously described.^30^ In summary, the method used for construction was bootstrapping using the software PASTA.^33^ The model inputs for creating an effective tree were described previously.^34^ In short, multiple iterations of tree creation were done, and the best model tree determined by a computed maximum likelihood was chosen to improve the quality of the tree.

### Secondary Structure Prediction

We predicted the RNA secondary structure through MaxExpect,^35–38^ a tool based on maximizing base-pair accuracy. Statistical learning predicts pair probability using a partition function calculation to determine the nucleotide’s base-pair vs single-stranded probabilities.^38^ The predictions were conducted using the Mathew lab’s RNAStructure Web Server.^36^

### Plasmid Construction

Plasmids generated in this study to transcribe and purify RNA variants were constructed using the pUC19 cloning vector. The restriction sites used in this plasmid were the *Bam*HI (5′-G∧GATCC-3′) and *Eco*RI (5′-G∧AATTC-3′) sites. Each riboswitch sequence was inserted between these two restriction sites. The T7 promoter sequence (5′-TAATACGACTCACTATAG-3′) was inserted between the riboswitch sequences and the *Bam*HI restriction site to allow for T7 RNA polymerase-mediated RNA transcription.^16,26,29,39^ These were ordered from Life Scientists’ Service Center and were sequenced upon arrival to confirm the correct construct.

### DNA and RNA Purification

DNA plasmids designed previously^16,26^ and those constructed as described above were prepared following the basic Qiagen midi-prep procedure as described previously,^16,26,29,39^ following the manufacturer’s protocol. Subsequent amplification of the target gene of interest was done via PCR with the Q5 High-Fidelity protocol (NEB) as previously published^26^ and filtered through the Monarch PCR and DNA Cleanup kit following the manufacturer’s protocol. PCR primers were designed to overlap with the restriction sites *Bam*HI and *Eco*RI to achieve a *T*_m_ of 61–65 °C (SI Table 2). *In vitro* RNA transcription was done up to overnight (16 h) with the T7 High Yield RNA Synthesis Kit (NEB) and cleaned with the Monarch RNA Cleanup Kit, following the manufacturer’s protocol. RNAs were analyzed on 8% denaturing acrylamide gel to confirm full length and nondenatured condition (SI Figure 6). DNA and RNA concentrations were determined using a Nanodrop 2000c Spectrophotometer. Before experimentation, RNAs were snap-cooled by heating to 95 °C for 2 min, followed by incubation on ice for 2 min.

### Fluorescent Probe Preparation

Riboglow probes Cbl-Cy5 and Cbl-4xGly-Atto590 were gifts from Amy Palmer at CU Boulder that were HPLC purified and characterized previously.^16^ They were diluted in phosphate-buffered saline (PBS) after being brought up in DMSO. These probes were characterized and used in the Riboglow platform previously.^16,26,29,39^ The concentration of the probes was determined via UV–vis absorbance with a Cary 60 UV–vis Spectrophotometer. The spectrophotometer was blanked with 1x PBS. Cbl-Cy5 and Cbl-4xGly-ATTO590 were diluted with 1x PBS in an 8 μL quartz cuvette. The concentration was determined using absorbance at 646 nm with an extinction coefficient of 271,000 L mol^–1^ cm^–1^ and absorbance at 594 nm with an extinction coefficient of 120,000 L mol^–1^ cm^–1^ for Cbl-Cy5 and Cbl-4xGly-ATTO590, respectively.^16^

### Fluorescent Induction Binding Assay

The binding affinity of the Cbl-based probes and the purified RNA tags was determined using fluorescence intensity, following the established principle that the fluorescence intensity of the probe alone is dim and increases substantially upon RNA binding.^16^ RNAs and probe were diluted in RNA buffer (100 mM KCl, 1 mM MgCl_2_, 10 mM NaCl, 50 mM HEPES, pH 8.0) and samples were loaded into a Corning 384-well black walled flat bottom microplate (with lid). Each well had a total volume of 30 μL, with 0.1 μM of Cbl-Cy5 and varying amounts of RNA to 4 μM per well (4000 nM, 2000 nM, 1000 nM, 500 nM, 250 nM, 125 nM, 62.5 nM, 31.25 nM, 15.63 nM, 7.81 nM, 3.9 nM, 0 nM). This range was selected based on the previously reported *K*_D_ of RNA tag variant “A-tag”/*env8* in the low nM range.^16,39^ All samples were prepared in an RNase-free hood, with careful attention to dilution calculations. After samples were loaded into the microplate, samples were incubated at room temperature in the dark for 40–60 min (plates were wrapped in aluminum foil) based on previous observations of Riboglow RNA-probe titrations.^26,29,39,40^ The fluorescence intensity of Cbl-Cy5 was measured using a BioTek Synergy H1 Microplate Reader (640 nm excitation, 670–700 nm emission, measured every 1 nm). Fluorescence intensity was gain corrected to the intensity of the highest RNA concentration well, and background intensity was subtracted from probe fluorescence values using buffer fluorescence values. Fluorescence values were integrated over all measured wavelengths. The corrected integrated fluorescence intensity values for each well were determined. The resulting values were normalized and fit to the nonlinear model as described by

$$Y=m-\left(n-m\right)\frac{(c+x+K)-\sqrt{{\left(c+x+K\right)}^{2}-\left(4\mathrm{cx}\right)}}{2c}$$

where *Y* is the corrected normalized fluorescence value, *m* is the lower baseline, *n* is the upper baseline, *c* is the probe concentration (nM), *x* is the RNA concentration, and *K* is the *K*_D_ (nM). Each variant tested was done with at least three biologically independent samples, along with a triplicate technical replicate for each.

### In Vitro Fluorescent Lifetime Imaging Microscopy (FLIM)

*In vitro* fluorescent lifetime measurements were collected for Cbl-Cy5 and Cbl-4xGly-ATTO590 in the absence and presence of purified RNA tag variants. All experiments were conducted in RNA buffer (100 mM KCl, 1 mM MgCl_2_, 10 mM NaCl, 50 mM HEPES, pH 8.0). Samples in the absence of RNA had a final probe concentration of 0.5 μM Cbl-Cy5 or 5 μM Cbl-4xGly-ATTO590, while samples including RNA variants had a final RNA concentration of 5 μM and a final probe concentration of 0.5 μM (Cbl-Cy5) or 5 μM (Cbl-4xGly-ATTO590). These conditions ensured that the concentration was well above the published *K*_D_ ^16,39^ for A-tag/Cbl binding. Samples were incubated for 40–60 min at room temperature in the absence of light. For each construct, at least three biologically independent samples were prepared with at least three technical replicates. Solutions (greater than 10 μL) were added to either a 35 mm μ-dish or a glass coverslip for FLIM acquisitions. Data was acquired as established previously^26,29,39^ using an Abberrior STED FLIM microscope with confocal capabilities, with a fixed imaging area of 512 × 512 pixels using a Picoquant Timeharp 260 card. Images were acquired until a total threshold of 10^4^ photon counts was reached with a pulsed laser of 40 MHz and excitation at 640 nm (Chroma 675/50 ET Bandpass filter) for Cbl-Cy5 or excitation at 561 nm (Semrock Em01-R488/568 + SP01–633RU filter) for Cbl-4xGly-ATTO590. Data was recorded using Picoquant SymPhoTime 64 software (v.1–9) and analyzed as described below.

### Multiexponential Reconvolution Fitting Analysis

Multiexponential reconvolution fitting of fluorescent decay measurements acquired using FLIM as described above was conducted using the Picoquant SymPhoTime 64 software with the calculated IRF as previously reported.^26,29,39^ Each pixel has a lifetime that is detected using Time-Correlated Single Photon Counting (TCSPC). The photon arrival times of each pixel were aggregated into a histogram of arrival times for each region of interest (ROI), defined as each *in vitro* acquisition (typically 512 × 512 pixels per acquisition). The subsequent decay function was further analyzed to extract fluorescence lifetime values. Multiexponential reconvolution fitting parameters were assessed as previously established, in which *in vitro* FLIM for Cbl-Cy5 was best fit using biexponential and *in vitro* FLIM for Cbl-4xGly-ATTO590 was best fit using triexponential reconvolution.^26,29,39^ The resulting amplitude-weighted average lifetime was assigned a false color scale for visualization.

## Results and Discussion

The fluorescence lifetime readout of Riboglow probes used in Riboglow-FLIM is sensitive to RNA sequences that bind, as we previously established for two proof-of-concept RNA variants, called the A-tag and D-tag (SI Table 1).^26^ To further expand the Riboglow platform systematically, we explored the RNA tag sequence space. We hypothesized that identifying several RNA sequence variants that affect Riboglow probe fluorescence lifetime will provide a foundation to design a suite of Riboglow-FLIM RNA tags for multiplexing. Two main methods were used to identify new RNA tag variant candidates: One, we used a literature-guided approach to collect RNA sequences that had either indirect or direct evidence of Cbl binding. Second, we rationally designed RNA sequence mutants based on existing biochemical evidence in the literature. We evaluated the binding of each RNA tag variant candidate to a model Riboglow probe and assessed fluorescence lifetime *in vitro*. Generating RNA tag variants and characterizing them in the context of the Riboglow platform may be used as the basis for designing multiplexed RNA tag detection with Riboglow-FLIM in the complex cellular environment in the future (Figure 1b).

### Literature-Guided RNA Tag Expansion Approach

Our first approach used to vary the RNA tag of the Riboglow platform is literature-guided where we collected RNA sequences that indicated RNA binding to Cbl indirectly^30,32,41^ or directly.^16,26,39^ We aligned the sequences showing indirect binding in the literature via a green fluorescent protein (GFP) gene expression repression assay^30^ and created iterations of phylogenic trees using PASTA,^33^ resulting in the maximum likelihood phylogenetic tree (Figure 2a).^34^ Both the rooted (Figure 2a) and unrooted (SI Figure 1) trees were evaluated to avoid bias. We selected six phylogenic variants (F–K tags, SI Table 1) based on these ancestral differences. For the greatest probability of achieving multiplexing capabilities, we chose sequences that covered the entire phylogenic tree. The second criterion for selecting RNA sequences was the assessment of indirect Cbl binding values through the GFP fold repression assay. We note that the GFP fold repression assay indicates evidence of Cbl-binding, but does not report on biochemical binding directly. To capture diverse sequences, we chose those with values on the high, middle, and low ends of documented fold repression values (Figure 2a).^30^ Together, the assessment of published RNA tag sequences through phylogenetic analysis yielded a series of RNA tags to evaluate biochemically.

**Figure 2:**
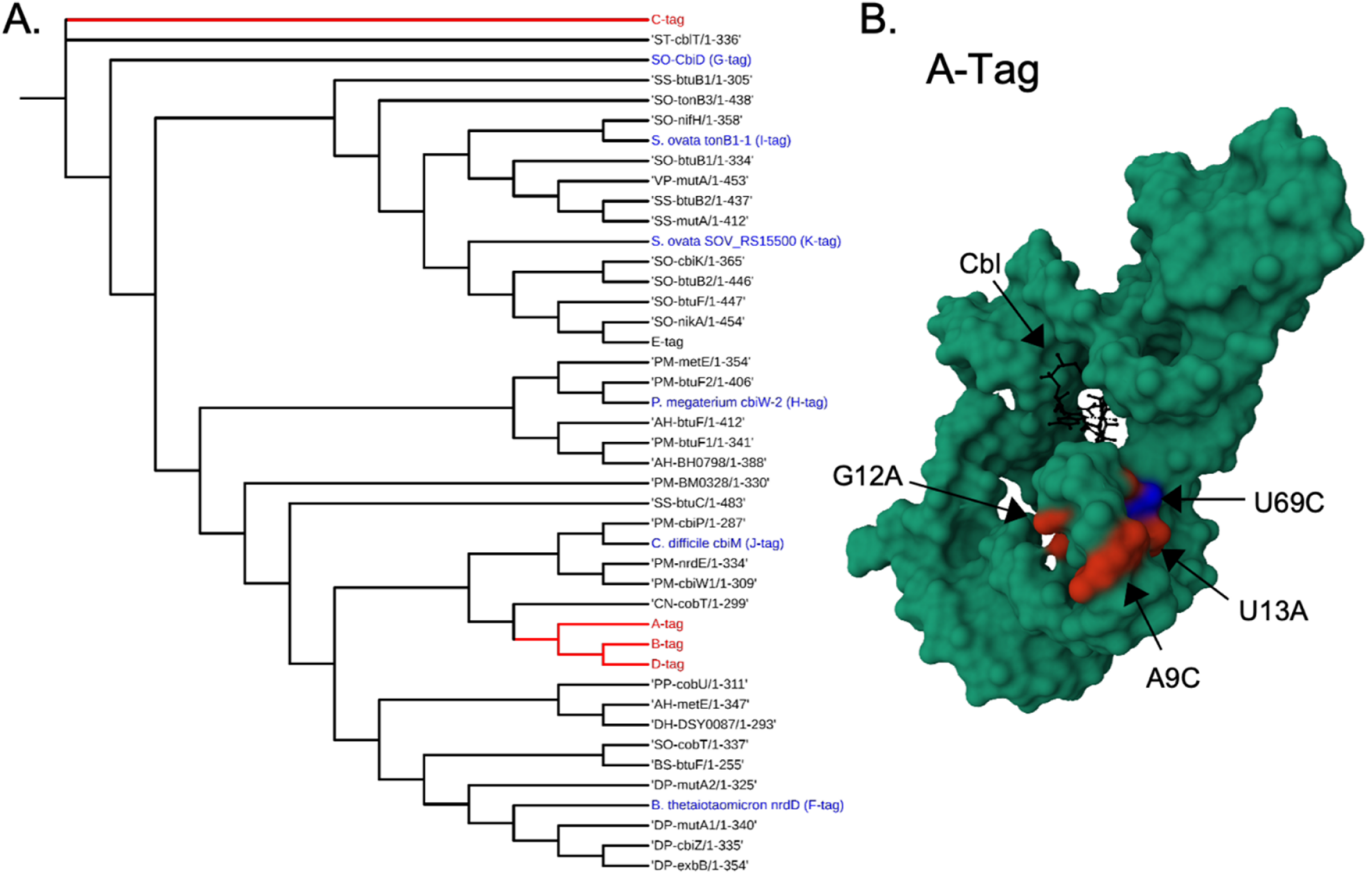
Methods of RNA tag sequence expansion. Two methods were used to find Cbl-binding RNA tag candidates, literature-guided (A) or rational mutations (B). (A) The phylogenic tree was developed using literature and known sequences of Cbl-riboswitches. Red: sequences that were previously characterized as variants of the Riboglow RNA tag (A–D tags).^16^ Blue: sequences were chosen from a phylogenic tree of RNA sequences with indirect evidence of Cbl-binding (F–K tags).^30^ (B) Rational point mutations of the parent Riboglow-tag, A-tag (PDB ID 4FRN)^27^ yield R-Tag (mutation U69C, blue), and Z-Tag (mutations A9C G12A U13A, red).

Along with indirect binding within the literature-guided approach, we identified RNA sequences showing direct binding to Cbl in the literature.^16,26,31,32,39,41^ Previously established Riboglow RNA tags evaluated through *in vitro* fluorescence-intensity-based measurements include the A-tag, B-tag, C-tag, and D-tag.^16^ The A- and D-tag were used in the context of Riboglow-FLIM *in vitro* and live cells for proof-of-concept lifetime-based measurements.^16,26,29^ Previously, the A- and D-tags were characterized by quantifying fluorescent intensity^16^ and fluorescent lifetime when binding the Cbl-4xGly-ATTO590 probe, but not Cbl-Cy5.^26^ In addition to the previously characterized Riboglow tags A-D *in vitro* in intensity-based measurements,^16,26^ we identified and assessed three additional RNA sequences because they were reported^31,32,41^ to directly bind to Cbl through biochemical evidence. One, we identified an RNA aptamer (here called the B12-tag, SI Table 1) with a published RNA crystal structure that includes a Cbl ligand.^31^ This sequence was only around 35 nucleotides long and is the shortest RNA in our series, compared to the model Riboglow tag (A-tag) of around 100 nucleotides in length.^16,26^ The second tag selected based on published biochemical evidence was an aptamer previously identified via *in vitro* mutagenesis.^32^ Through eight rounds of selection from an original pool of 5 × 10^14^ RNA sequences, this sequence was the majority product (called the S-tag here, SI Table 1).^32^ The third RNA sequence with biochemical evidence of Cbl-binding in the literature was a conserved structure related to regulating vitamin B-12 (Cbl) synthesis (called the E-tag here, SI Table 1).^41^ Together, our phylogenetic approach to identify Cbl-binding RNA tag candidates and in-depth search of available RNA sequences in the literature that have biochemical evidence of Cbl-binding led us to assemble 13 different RNA tag sequences for further fluorescence lifetime characterization in the context of Riboglow-FLIM.

### Rationally Designed Mutations for RNA Tag Expansion Approach

Our second approach to assembling RNA sequence variants as Riboglow-tag candidates is using rational mutations. For the greatest possibility of identifying RNA tags with lifetime differences, we used the most thoroughly characterized Riboglow RNA tag as the parent, the A-tag/*env8*.^16,26,29,39,40^ We systematically introduced mutations by three approaches: nucleotide deletion, nucleotide insertion, and point mutations. We aimed to purify the resulting RNA tag candidates as before and perform fluorescence lifetime assays analogous to tags A-D, E-K, S, and B12 for direct comparison (Figure 2).

To design RNA tag variants through nucleotide deletion, we considered a previous observation that truncating the 3′-end of the A-tag contributes to a change in fluorescence intensity turn-on versus the full-length A-tag.^16^ Because previous studies assessed fluorescence intensity (but not fluorescence lifetime), we hypothesized that deleting a similar region in the context of the A-, B-, C-, and D-tag (SI Table 1)^16^ may alter fluorescence lifetime (SI Figure 2). We, therefore, explored these four deletion sequences further, indicating each truncation with the “t” in the tag name.

In addition to deletion mutations, we rationally introduced insertion mutations in the parent A-tag sequence. We added G-C pairs, hypothesizing that these may allow for stronger Watson–Crick interactions of distant nucleotides (SI Figure 2). To select sites of nucleotide insertions, we first predicted the RNA secondary structure of the A-tag through MaxExpect.^35–38^ The predicted secondary structure (SI Figure 2) revealed stem areas or areas where base-pair interactions exist. These predicted interactions align with interactions observed in the Cbl/RNA A-tag structure.^25^ We introduced G-C pairs on either end of the predicted stem pairs to increase the area of stems with G’s and C’s, resulting in the N-tag (SI Figure 2, SI Table 1).

Lastly, we introduced point mutations in the wild-type RNA A-tag, yielding the Z- and R-tag, and Atmut-tag (SI Figure 2, SI Table 1). These mutations were introduced rationally based on the following considerations. Two areas in the A-tag structure^16,42^ participate in long-distance interactions,^42^ nucleotides 9–13 and 69–70 (Figure 2b, SI Figure 2). These interactions are caused by nonsequential nucleotides. The mutation in the R-tag (nucleotide 69, blue in Figure 2b, U69C) is a point mutation, whereas the Z-tag is mutated at three nucleotides (Figure 2b, red, A9C G12A U13A). This study focused on how long-distance interactions between nonsequential nucleotides influenced lifetime readout rather than their overall interaction strength, therefore we aimed to maintain interaction strength for the R- and Z-tag. The Atmut tag has previously shown no fluorescent turn-on (showing an inability to dequench the probe), hence this variant serves as a nonbinding variant.^16^ A series of RNA variants with point mutations, insertion mutations, and deletion mutations that yielded seven novel sequences were designed for *in vitro* FLIM characterization.

### Characterization of Literature-Guided RNA Variants

For characterizing the previously studied Riboglow tags through our Riboglow-FLIM *in vitro* workflow, we first confirmed binding of the A- and D-tag RNAs to Cbl-Cy5 (SI Figure 4, Table 1). The binding affinity of Cbl to the RNA A-tag (*K*_D_ = 73 ± 11 nM) and the RNA D-tag (*K*_D_ = 9.5 ± 1.6 nM) was in the nM range as determined by a fluorescent inductive binding assay in our hands, in line with published literature values (Table 1, SI Figure 4).^16,39,40^ Considering these values, *in vitro* FLIM experiments were set up such that the concentrations of RNA (5 μM) and probe (500 nM) were at least five times above the binding affinities. Given the *K*_D_ for these experiments in the nM range, these conditions and the incubation for at least 1 h prior to data collection were used to achieve equilibrium.^43^ To quantify the fluorescent lifetime of Cbl-Cy5 in the presence of the RNA A-tag, B-tag, C-tag, and D-tag, fluorescence decay curves (Figure 3a) were fit by multiexponential reconvolution. We have previously found that multiexponential reconvolution with parameters *n* = 2 resulted in the best fit for the average amplitude-weighted lifetime for Cbl-4xGly-ATTO590 ^26,39^ and Cbl-Cy5, see defined in the methods.^29,39^ The average lifetime values quantified this way indicated fluorescence lifetime turn-on for all four tags (Figure 3b). The average lifetime of the A-tag RNA (1.01 ns), B-tag RNA (1.06 ns), and D-tag RNA (1.01 ns) was significantly higher than the lifetime of the quenched probe (0.57 ns). However, we found the C-tag RNA (0.61 ns) only showed a small lifetime increase (Figure 3b). Interestingly, although these four tags had a fluorescent lifetime increase, only three are differentiable from each other (Figure 3b). The A-tag and D-tag RNAs result in similar lifetimes in the presence of Cbl-Cy5 (1.007 ± 0.004 ns for A-tag and 1.012 ± 0.004 ns for D-tag).

**Table 1:** Summary of Binding Affinities of Different RNAs in the Presence of Cbl, or Cbl-Derived Probes

name of RNA Tag	name of Probe	K_D_	source
A-Tag	Cbl	99 ± 30 nM	Sarfraz et al.^39^
		37 ± 1 nM	Braselmann et al.^16^
	Cbl-5xPeg-ATTO590	34 ± 9 nM	Braselmann et al.^16^
		28 ± 7 nM	Lennon et al.^40^
	Cbl-Cy5	73 ± 11 nM	this study.
D-Tag	Cbl	2.2 ± 1.6 nM	Braselmann et al.^16^
	Cbl-5xPeg-ATTO590	3.0 ± 0.6 nM	Braselmann et al.^16^
	Cbl-Cy5	9.5 ± 1.7 nM	this study
F-Tag	Cbl-Cy5	>4000 nM	this study
Atmut-Tag	Cbl-Cy5	>4000 nM	this study

**Figure 3:**
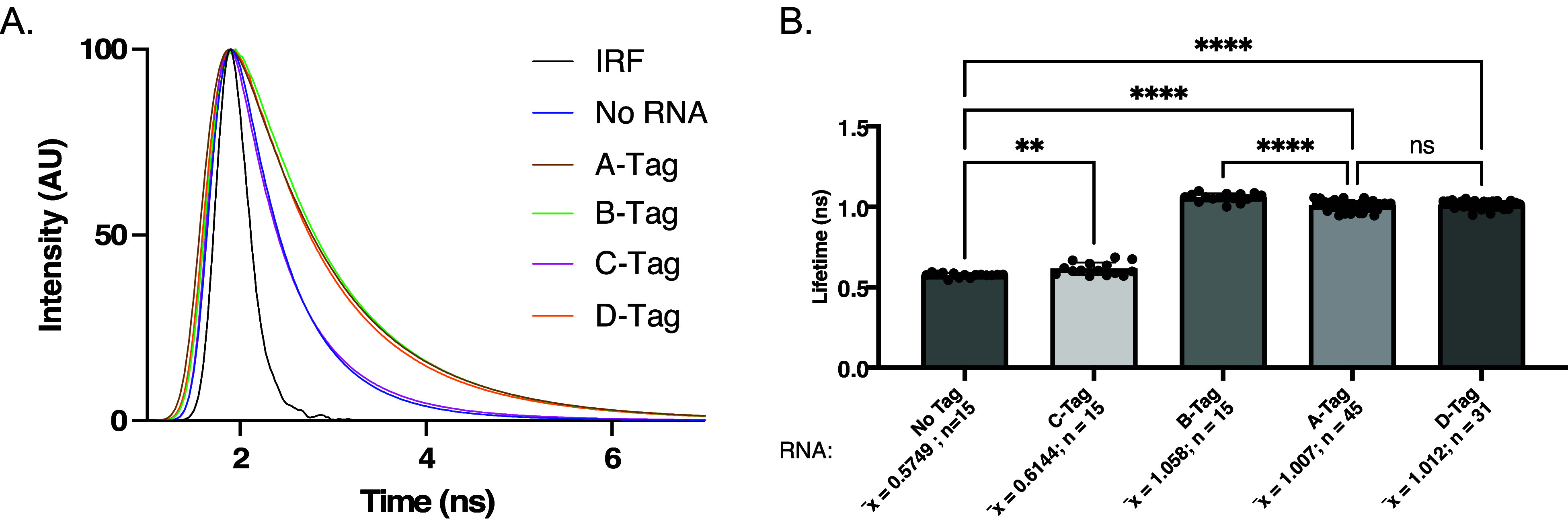
Fluorescence lifetime of Riboglow RNA tags in the presence of Cbl-Cy5. (A) Representative lifetime decay curve for tags A-D. The IRF was used to convolute the decay curves. Control with the Cbl-Cy5 probe alone: dark blue (“No RNA”). (B) Average fluorescent lifetime values after fitting curves in (A) by multiexponential reconvolution with *n* = 2 parameters. Each point represents one acquisition. Experiments were repeated at least 3 independent times with at least 3 technical replicates, n: number of acquisitions, p-values listed (ns: *p* ≤ 0.5; *p ≤ 0.05; **p ≤ 0.01; ***p ≤ 0.001; ****p ≤ 0.0001). One-way ANOVA (95% confidence limit); post hoc test (Tukey HSD). Error bars indicate mean and standard deviation (±SD).

Next, we characterized sequences with direct binding evidence to Cbl (the RNA E-tag, S-tag, and B12-tag) by quantifying the fluorescent lifetime of Cbl-Cy5 in the presence of these RNA tags using our established *in vitro* FLIM workflow (Figure 4a). The quantified lifetime values for E-, S-, and B12-tag (0.56, 0.57, and 0.58 ns, respectively, Figure 4b) were comparable to that of the free Cbl-Cy5 probe (0.57 ns, Figures 3a and 4a), indicating no fluorescence lifetime turn-on. These findings led us to conclude that direct binding evidence may not necessarily correlate to Riboglow-probe fluorescence turn-on.

**Figure 4:**
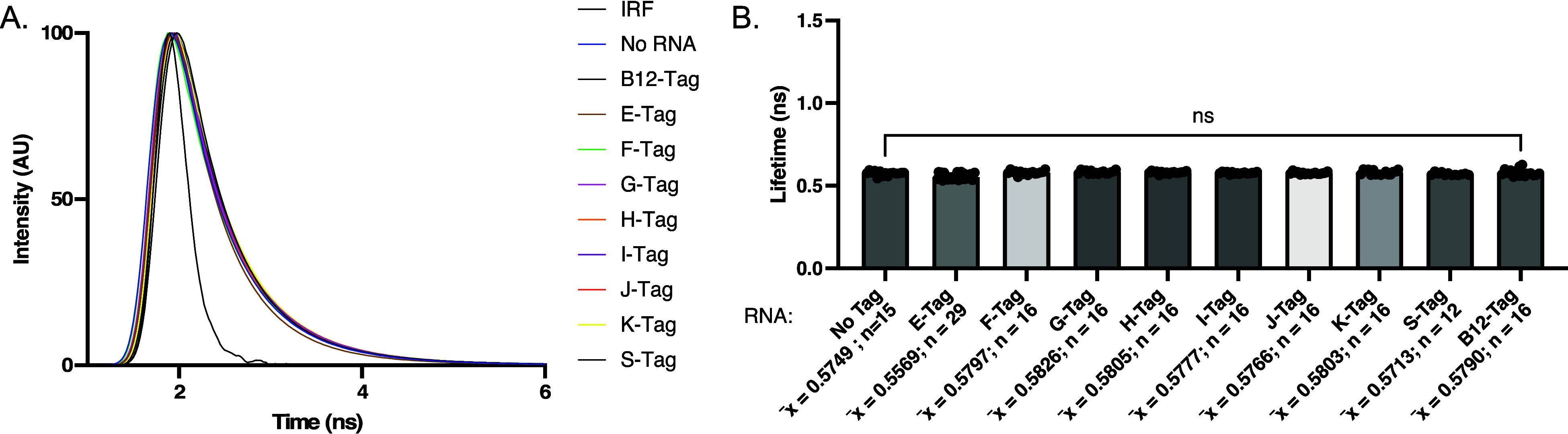
Fluorescence lifetime of literature-guided purified RNA tags in the presence of Cbl-Cy5. (A) Representative lifetime decay curve for RNA tags chosen by phylogeny and literature. The IRF was used to convolute the decay curves. Blue: Cbl-Cy5 probe alone (“No RNA”). (B) Average fluorescent lifetime values using multiexponential reconvolution fitting of decay curves in (A) with *n* = 2 parameters. Experiments were repeated at least 3 independent times with at least 3 technical replicates, n: number of acquisitions, p-values listed (ns: *p* ≤ 0.5; *p ≤ 0.05; **p ≤ 0.01; ***p ≤ 0.001; ****p ≤ 0.0001). One-way ANOVA (95% confidence limit); post hoc test (Tukey HSD). Error bars indicate mean and standard deviation (±SD).

For indirect binding evidence, phylogeny resulted in six different sequences (RNA tags F–K, SI Table 1). Similarly to previously described^26,29,39^ and established here (Figures 2 and 3), the fluorescent lifetime of Cbl-Cy5 in the presence of the RNA tags was quantified via multiexponential reconvolution (*n* = 2) (Figure 4a,b). All RNA tags identified through phylogeny had a lifetime comparable to free Cbl-Cy5 (0.57 ns), indicating there was no fluorescence turn-on in the presence of these RNAs. We assessed probe binding to the F-tag as a representative sample for tags with no fluorescence turn-on using a fluorescence induction binding assay (Table 1, SI Figure 4) and indeed observed no probe binding (*K*_D_ > 4000 nM) compared with readily observed binding of the A-tag and D-tag RNAs in this assay. The lifetimes and binding assay values indicate that an extensive literature search and phylogenetic determination may not be fruitful for systematic Riboglow tag RNA expansion.

Exploring a literature-guided approach using phylogeny for further selection did not yield RNA sequences that produced differentiable fluorescence lifetimes. We concluded that RNA sequences cataloged as phylogenetic variants in the literature do not yield probe fluorescence lifetime changes, likely because no binding to our Riboglow probe occurred, as represented by binding titration of RNA F (Figure 4). It is noteworthy to emphasize that the β-axial ligand of what is commonly referred to as “Cobalamin” in different studies may vary. Our Riboglow platform uses Cbl derivatives that uniformly have CN- as the β axial ligand.^16^ However, the identity of the β-axial ligand for Cbl affects binding properties for different RNA riboswitch sequence classes, and some RNA sequences may not bind to CN-Cobalamin.^44^ This may lead to ambiguity for the purposes of our literature search.

The literature-guided approach of sequences directly indicating Cbl binding led us to investigate the B12-tag, where RNA-binding to Cbl was reported.^31^ Importantly for our work, the crystal structure of the B12-tag bound to Cbl clearly shows CN as the β-axial ligand,^31^ the same β-axial ligand as in our Riboglow probes. However, we did not observe fluorescence lifetime increase in the Riboglow probe, despite previously observed binding with nM affinity *in vitro*.^31^ Further analysis of the crystal structures of both A-tag^27^ (where the Riboglow probe is dequenched) and the B12-tag^31^ (where the Riboglow probe is not dequenched) show different degrees of contact between the RNA and Cbl. The A-tag RNA surrounds most of Cbl, whereas the B12-tag RNA surrounds only a partial area of Cbl. This difference in coverage could result in the observation that the Riboglow probe remains quenched even when the RNA B12-tag is bound, because the fluorophore and Cbl may remain in sufficient contact and/or interact in space and therefore remain quenched.

### Characterization of RNA Variants with Rational Mutation

Next, we characterized RNA tags designed through rational mutations using insertions, deletions, and point mutations based on the previously studied Riboglow A-, B-, C-, and D-tags.^16^ The lifetime of the previous Riboglow tags showed an increase in lifetime versus the probe alone (Figure 3b). We quantified the effects of the rationally designed mutations on the lifetime with our fluorescent lifetime assay, as for the other mutations (Figure 5a). Among the designed RNA mutants, we observed fluorescence lifetimes ranging from that of free Cbl-Cy5 (∼0.5 ns) to full turn-on (∼1.1 ns) (Figure 5b).

**Figure 5:**
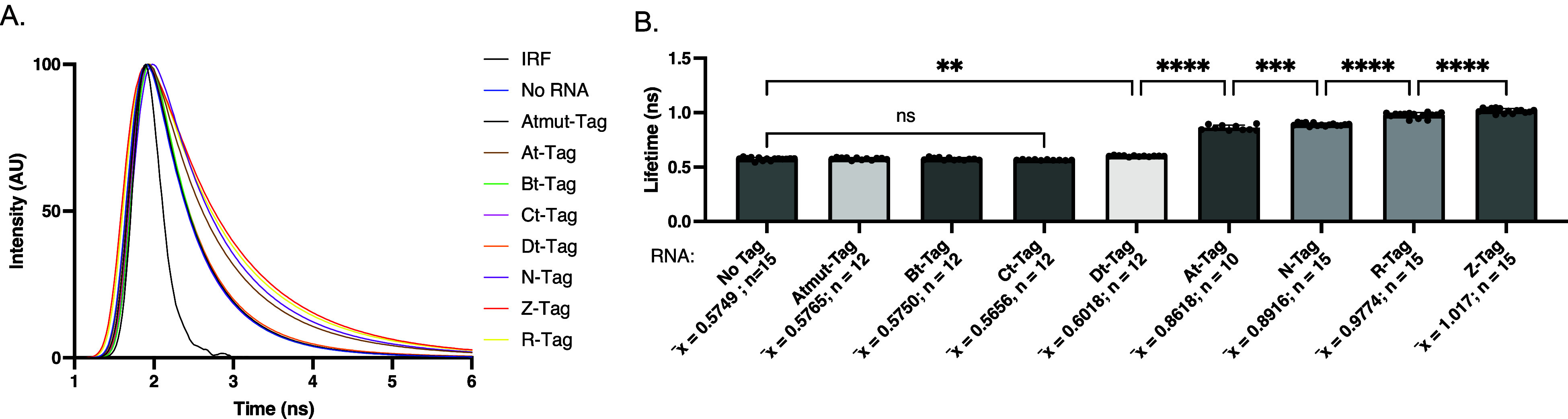
Fluorescence lifetime of rationally designed purified RNA tags in the presence of Cbl-Cy5. (A) Representative lifetime decay curve for tags with rational mutations. The IRF was used to convolute the decay curves. Blue: Cbl-Cy5 probe alone (“No RNA”). (B) Average fluorescence lifetime values using multiexponential reconvolution fitting of decay curves (i.e., (A)) with *n* = 2 parameters. Each point represents an acquisition repeated for at least 3 independent experiments with at least 3 technical replicates, with p-values listed (ns: *p* ≤ 0.5; *p ≤ 0.05; **p ≤ 0.01; ***p ≤ 0.001; ****p ≤ 0.0001), n: number of acquisitions. One-way ANOVA (95% confidence limit); post hoc test (Tukey HSD). Error bars indicate mean and standard deviation (±SD).

For the deletion mutations where the tags were truncated, the lifetime values were either low or similar to those of the Cbl-Cy5 probe alone (Figures 3–5). Truncation of B-tag, D-tag, and Amut-tag all had lifetime values like that of free Cbl-Cy5, indicating no dequenching occurred. Atmut had both truncation and mutation in the A-tag which eliminated binding of the probe to the RNA.^16^ Therefore, no fluorescence intensity-based dequenching was observed before,^16^ in line with our observations. To confirm this finding, the binding affinity of Atmut to Cbl-Cy5 was determined via fluorescent inductive binding assay (SI Figure 4). Similarly to the F-tag, the Atmut-tag with Cbl-Cy5 resulted in an undetectable fluorescence increase (*K*_D_ > 4000 nM), indicating no probe binding (Table 1). Using these two binding assays, we hypothesized that no probe binding leads to no detectable fluorescence lifetime turn-on. Previous characterization of the At-tag resulted in a significant increase in fluorescence intensity^16^ and lifetime (Figure 5, 0.86 ns). Dt-tag also yielded a lifetime increase; however, the lifetime was lower than that for the At-tag (Figure 5, 0.60 ns). Together, when we evaluated the RNA tag deletion variants, we found that only the At- and Dt-tag resulted in an increase in fluorescence as opposed to the deletions in the Bt- or Ct-tag.

For RNA tags with insertion and point mutations, we observed more variable changes in the fluorescence lifetime of the probe than the truncated forms. N-tag has insertions (SI Figure 2) within the stems compared with the A-tag and resulted in a probe lifetime value of 0.89 ns. RNA R-tag and Z-tag yield lifetime values of 0.97 and 1.01 ns in the presence of Cbl-Cy5, respectively (Figure 5b). From characterizing rational mutations of previously used Riboglow tags, we found that deleting regions that align with truncations of the previous Riboglow tags lowered (Dt-tag) or completely removed the ability to alter the fluorescence lifetime of the probe (Figure 5b). Hence, we conclude that the RNA regions removed in variants Bt and Ct must be crucial to modulate probe fluorescence lifetime.

Rational mutations of our parent RNA A-tag resulted in RNA tags with different lifetime values. Overall, we identified regions of the A-tag that affect fluorescence lifetime changes (Figure 2b, SI Figure 2). These regions are related to the riboswitch’s ligand selectivity and could allow for more Cbl-fluorophore interactions and therefore affect quenching.

### Implications for Multiplexed RNA Imaging

With this study, we identified nine different RNA sequences that fulfill the criteria for enabling multiplexed RNA imaging in a live-cell environment, namely, tight binding to Cbl, and differentiable fluorescence lifetimes. These sequences were identified from the literature-guided (A-, B-, C-, and D-tags) and the rational mutation approaches (At-, Dt-, N-, R-, and Z-tags).

The RNA tags used in this study ranged from 35 to 300 nucleotides in length, with no discernible trend in lifetime values observed as nucleotide length increased. The well-established MS2 system has a total length of 456 nucleotides and each stem loop may bind to dimers of the binding protein fused to a fluorescent protein, yet the MS2 platform has been shown to not necessarily have adverse effect on live-cell imaging.^21^ Similarly, our previous work demonstrated that RNA tags of varying lengths did not interfere with live-cell imaging.^26^ All nine RNA tags developed in this study are unlikely to impact live-cell imaging due to the fact that they are similar in size as previous Riboglow RNA tags and significantly shorter than the validated MS2 system.

Previously, we have seen that different Riboglow probes have various fluorescence properties.^39^ Some of these properties include different fluorescence lifetime dynamic ranges, or the range between the absolute lowest and absolute highest fluorescent lifetime values achievable. One such example is the Cbl-4xGly-ATTO590 probe with a dynamic range of ∼ 3 ns. The difference in Cbl-Cy5 and Cbl-4xGly-ATTO590 dynamic ranges can be attributed to the fluorescent dye used, Cy5 vs ATTO590. Cbl-4xGly-ATTO590 quenching is in line with Förster resonance energy transfer, whereas Cbl-Cy5 is likely not,^16^ leading to varying ability of differentiation between different RNA species. To further investigate the dynamic range for our nine tags developed in this study, the fluorescence lifetime of three model RNAs (A-, D-, and N-tag) was assessed with Cbl-4xGly-ATTO590 (SI Figure 5). Observations from A-tag, D-tag, and no RNA tag are in line with previously published data.^26,39^ The fluorescence lifetime of Cbl-4xGly-ATTO590 in the presence of the N-tag RNA is lower than that in the presence of the A- and D-tag but higher than no RNA tag, a trend that is in line with Cbl-Cy5 (Figures 3,5). The difference in fluorescent lifetimes between A- and D-tag is comparable than the difference between D- and N-tag, ∼ 0.3 ns difference (SI Figure 5). Since A- and D-tag have shown the ability to track two RNAs simultaneously,^26^ we therefore hypothesize that D- and N-tag will be able to track RNAs simultaneously. This will increase our ability to track at least three RNAs simultaneously. Utilizing varying Riboglow components, such as different RNA tags and different fluorescent probes, we propose to tailor our ability to multiplex various RNAs simultaneously.

## Conclusions

With the need to further understand RNA dynamics, tools necessary for RNA visualization are critically needed. To differentiate multiple RNA species simultaneously, it is vital to have tools available for developing multiplexing imaging in live cells. One such example of a tool is the Riboglow-FLIM platform. Here, we systematically evaluated methods for expanding the Riboglow tag sequence space to identify and catalog RNA tag sequence variants for multiplexing capabilities and begin understanding underlying principles that allow for rationally designing RNA sequence variations to modulate Riboglow probe fluorescence. Two methods were investigated, namely literature-guided and rational mutations. While the literature-guided approach entailed biochemical evidence indicating either direct or indirect binding with Cbl, only four RNAs bound the Cbl-based fluorescence probe (Figure 3). The rational RNA mutations showed great promise in developing differentiable lifetime readouts, resulting in five different RNA tags (Figure 5). The lifetime differences of these tags can be further increased via utilizing various Riboglow probes, such as Cbl-4xGly-ATTO590 (SI Figure 5). We found that the nine tags developed here show great promise in increasing multiplex capabilities of RNA visualization in live cells with our Riboglow platform.

## Supplementary Material



## Notes

Figure S1: Unrooted phylogenic tree; Figure S2: Secondary structure predictions of RNA tags; Figure S3: Representative fluorescence lifetime imaging microscopy (FLIM) acquisitions; Figure S4: Fluorescence inductive binding assay for RNA tags; Figure S5: Fluorescence lifetime of RNA tag with Cbl-4xGly-ATTO590; Figure S6: Representative 8% Acrylamide RNA Gel; Table S1: Sequences of the RNA tags used in this study; Table S2: Primers used in PCR for RNA tag amplifications (PDF)
