# Supplementary material for: Expanding the Riboglow-FLIM Toolbox with Different Fluorescence Lifetime-Producing RNA Tags
Source: Biochemistry. 2025 May 15;64(11):2429–38. doi: 10.1021/acs.biochem.4c00567 (PMC12138973; doi:10.1021/acs.biochem.4c00567)
Supplement: Supplementary file 1 [file bi4c00567_si_001.pdf]

**Supplementary Information**

# Expanding the Riboglow-FLIM Toolbox with Different Fluorescence Lifetime-Producing RNA Tags

Zachary Stickelman, Nadia Sarfraz, Morgan K. Rice, Ben J. Lambeck, Sonja Milkovich, Esther Braselmann*

Department of Chemistry, Georgetown University, Washington, DC 20057

**Supplementary Figure 1:** Unrooted phylogenetic tree

**Supplementary Figure 2:** Secondary structure predictions of RNA tags

**Supplementary Figure 3:** Representative fluorescence lifetime imaging microscopy (FLIM) acquisitions

**Supplementary Figure 4:** Fluorescence inductive binding assay for RNA tags

**Supplementary Figure 5:** Fluorescence lifetime of RNA tag with Cbl-4xGly-ATTO590

**Supplementary Figure 6:** Representative 8% Acrylamide RNA Gel

**Supplementary Table 1:** Sequences of the RNA tags used in this study

**Supplementary Table 2:** Primers used in PCR for RNA tag amplifications

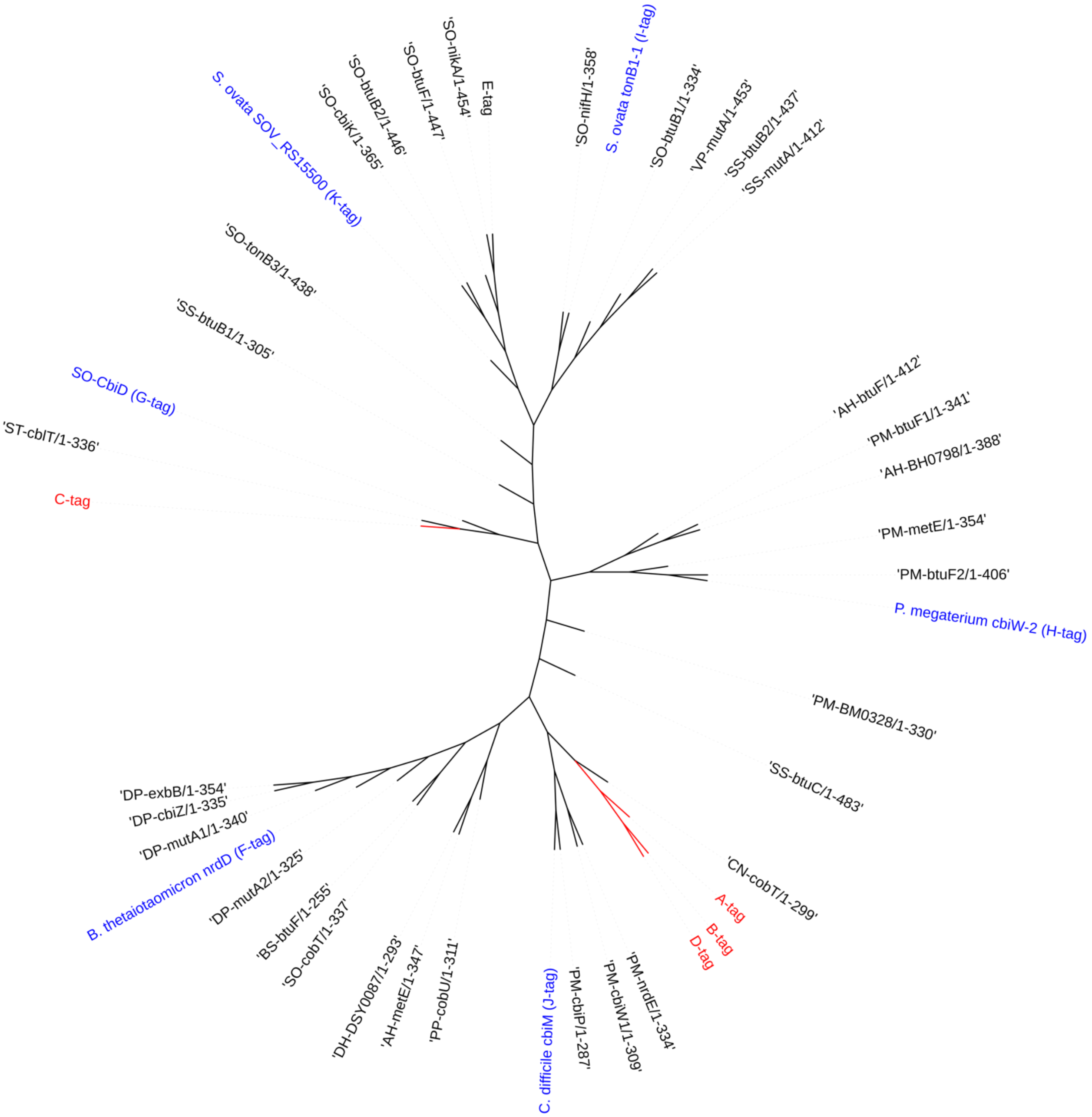


**Supplementary Figure 1:** Unrooted phylogenetic tree. Alignment of literature-guided sequences (see also Figure 2A for colors and names). Blue: RNA sequences from a green fluorescent protein assay based on phylogeny.[1] Red: previously characterized Riboglow RNA tags.[2–5]

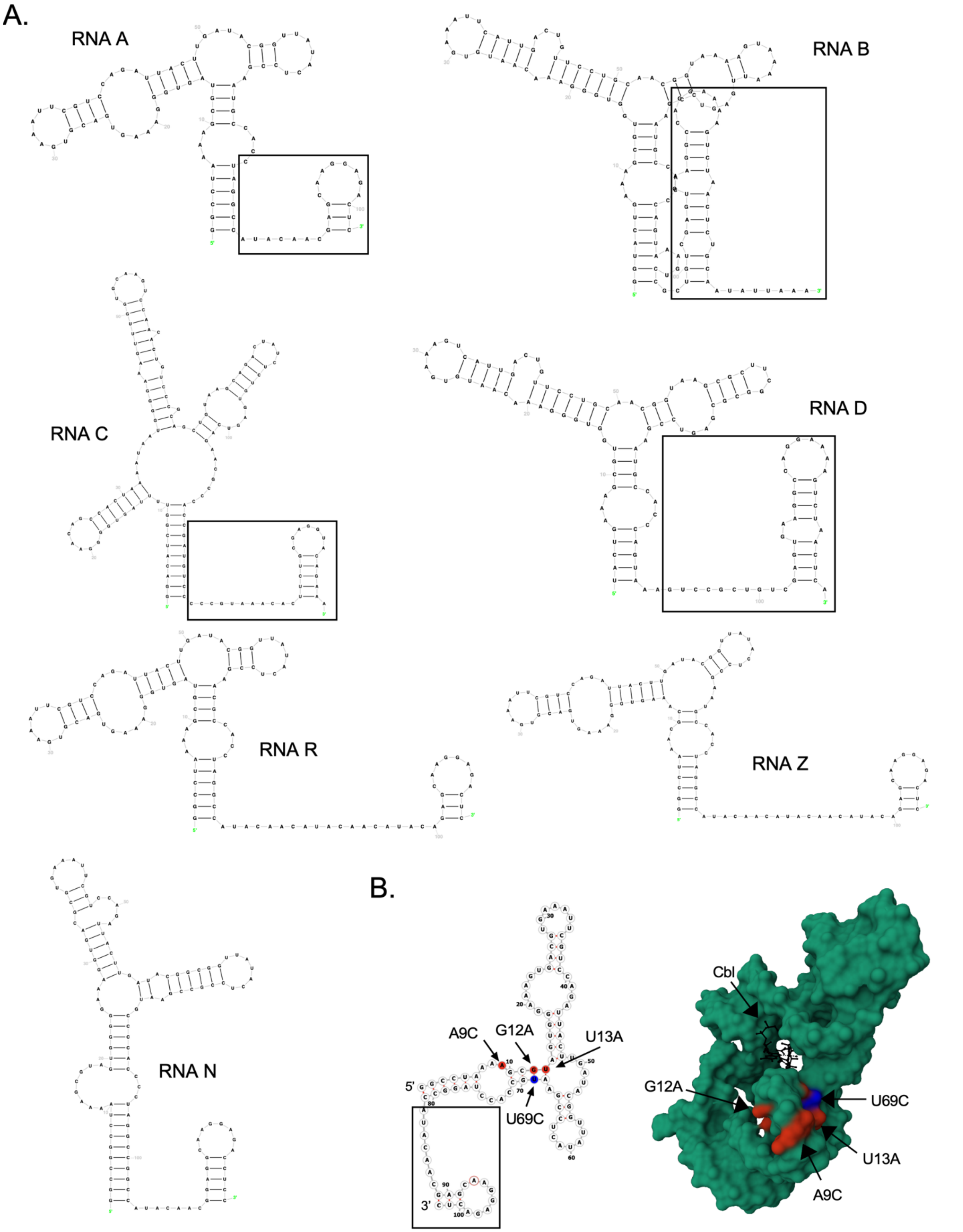


**Supplementary Figure 2:** Secondary structure predictions of RNA tags. (A) RNA secondary structures were predicted using MaxExpect[6–8] through the Mathews lab Webserver[9]. The boxed regions were

nucleotides cut from A-, B-, C-, and D-tag to achieve the truncated forms. (B) The predicted secondary structure and crystal structure (PDB ID 4FRN)[10] for the A-tag. Z-tag and R-tag mutations of A-tag: red (mutations A9C, G12A, U13A) and blue (mutation U69C).

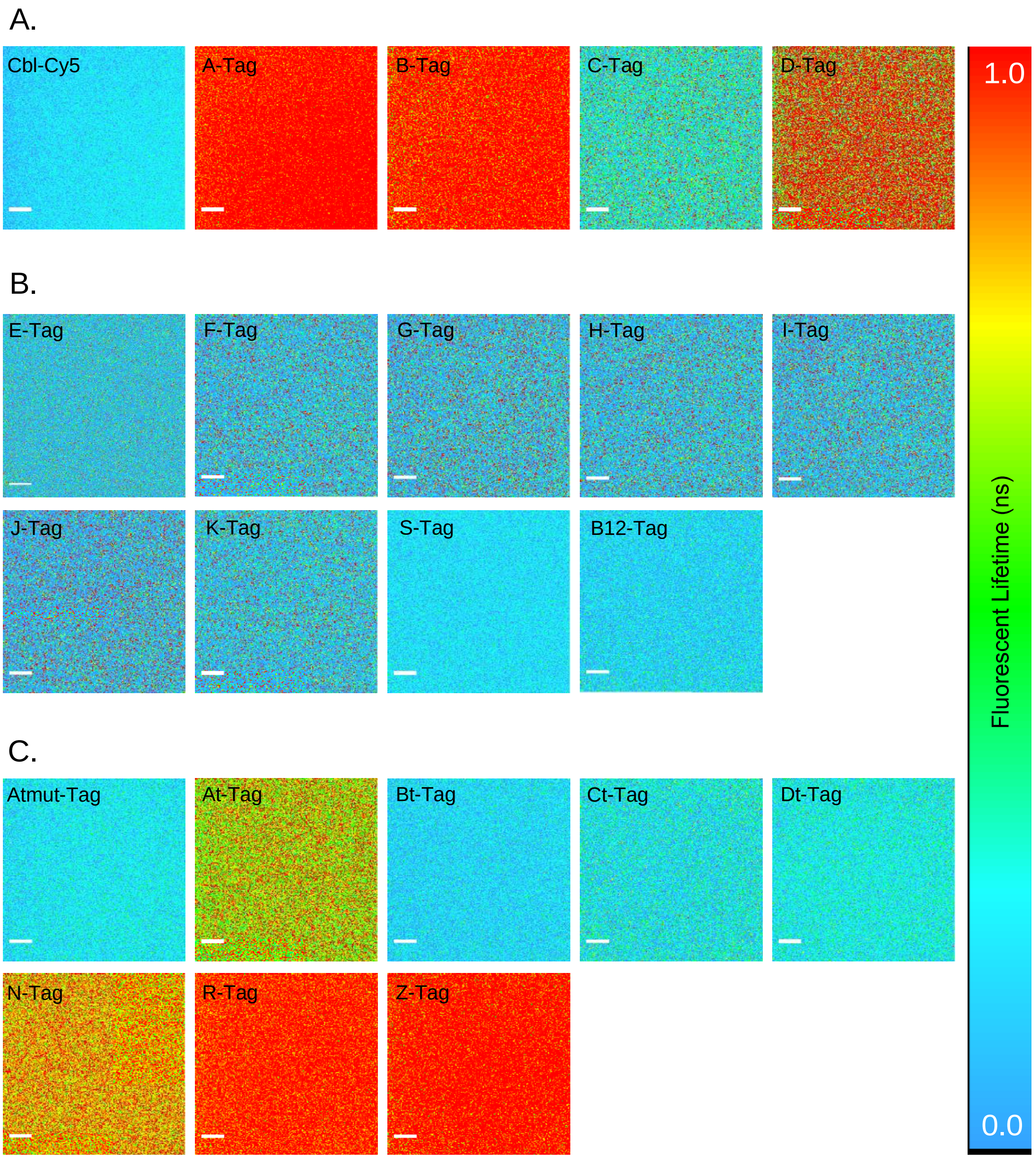


**Supplementary Figure 3:** Representative Fluorescence Lifetime Imaging Microscopy (FLIM) acquisitions. Fluorescence lifetimes were acquired by dropping a sample of probe + RNA on a cover slip and imaged by FLIM. The false-color scale (right) represents fluorescence lifetime values. Each pixel in each acquisition is assigned a false color associated with a lifetime value. (A) FLIM acquisitions of Cbl-Cy5 in the presence of previously studied Riboglow tags, as indicated. (B) FLIM acquisitions for Cbl-Cy5 in the presence of RNA tags obtained by literature-guided approach. (C) FLIM acquisitions of Cbl-Cy5 in the presence of rationally mutated RNA tag variants. "Cbl-Cy5" in (A) is the control condition representing the probe alone. Fluorescence lifetimes from these acquisitions are fit and reported in **Figure 3-5**. Scale bar = 50 µm.

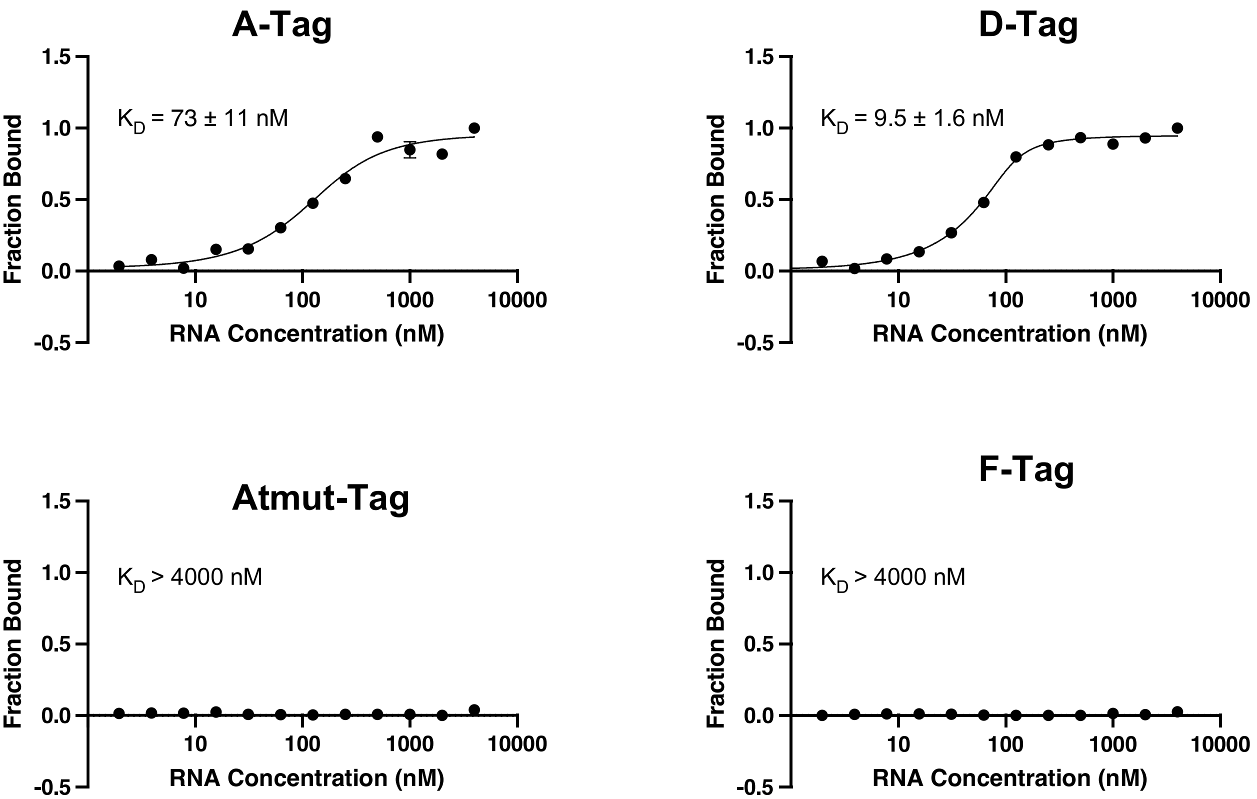


**Supplementary Figure 4:** Fluorescent inductive binding assay for RNA tags. Characterization of RNA / probe binding using fluorescent intensity. For each purified RNA tag, three biologically independent experiments with three technical replicates were conducted with the error being between replicates. The fluorescence was normalized to a probe sample without RNA and fit using the nonlinear model described in the methods. The highest RNA concentration used was 4000 nM, therefore the $K_D$ of RNAs for those not showing fluorescence increase here were estimated to be higher than this value.

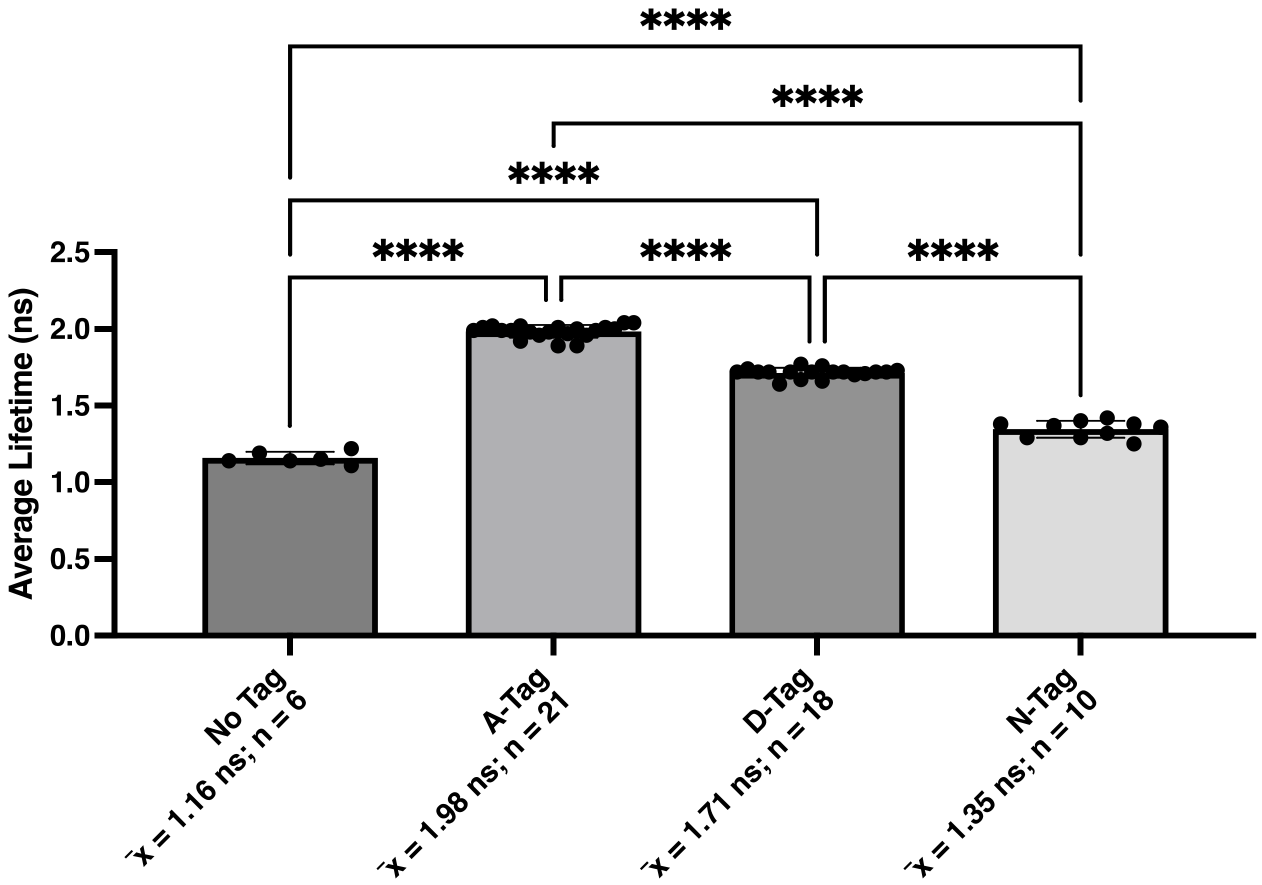


**Supplementary Figure 5:** Fluorescent lifetime of RNA tag with probe Cbl-4xGly-ATTO590. Average fluorescent lifetime values using multiexponential reconvolution fitting with *n=3* parameters. Each point represents an acquisition repeated with at least 3 independent experiments with p-values listed (ns: $p \leq 0.5$; $^{*}p \leq 0.05$; $^{**}p \leq 0.01$; $^{***}p \leq 0.001$; $^{****}p \leq 0.0001$). One-way ANOVA (95% confidence limit); post hoc test (Tukey HSD). Error bars indicate mean and standard deviation (±SD).

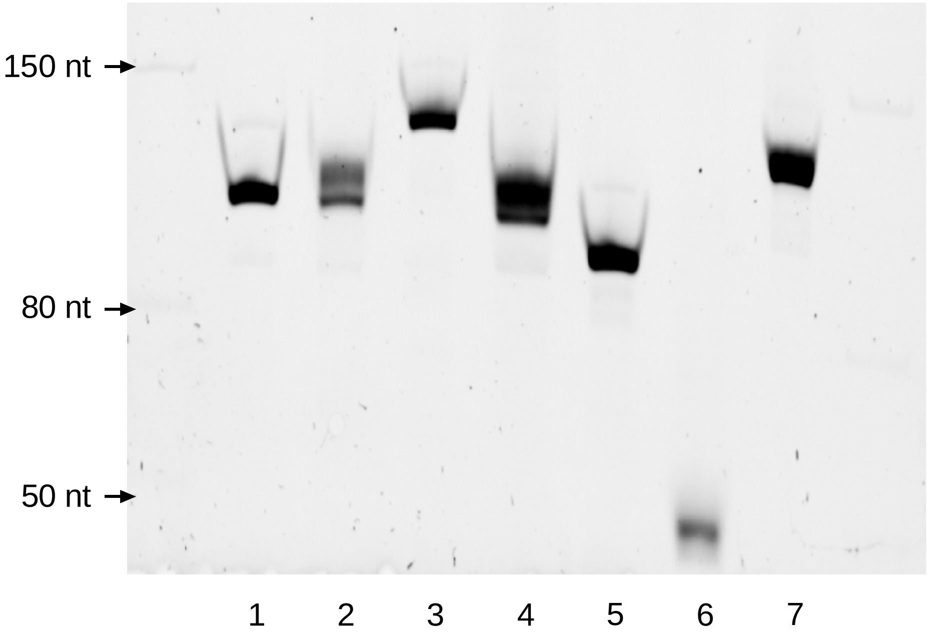


**Supplementary Figure 6:** Representative 8% Acrylamide RNA Gel. Analytical RNA gel used to determine size and quality of RNA post transcription / cleanup. Low range ssRNA ladder is on either sides of the seven well samples with related sizes of 50, 80, and 150 nt length. The samples represented by the seven wells are 1 = RNA At, 2 = RNA Bt, 3 = RNA Ct, 4 = RNA Dt, 5 = RNA Atmut, 6 = RNA B12, 7 = RNA R.

**Supplementary Table 1:** Sequences of the RNA tags used in this study. These were the sequences purified for *in vitro* measurements.

| Name | Rational/Origin | Sequence (5'→3') |
|---|---|---|
| A-Tag | Literature-guided: Direct Cbl binding; Crystal Structure (PDB ID 4FRN)[10] | GGCCUAAAAGCGUAGUGGGAAAGUGACGUGAAAUUCGUCCAGAUU ACUUGAUACGGUUAUACUCCGAAUGCCACCUAGGCCAUACAACGA GCAAGGAGACUC |
| B-Tag | Literature-guided: Direct Cbl binding; Crystal Structure (PDB ID 4FRN)[10] | GGUACUGAAAGCGUGGUGGGAAACAAUGUGAAAUUCAUUGACUGU UCCUGCAACGGUAAAAGUAAAAUUGAGUCCGAAUGCCACCCAGUA AAGUCCGCUGUCGAGUGAAGGCCAGGAAAAGUCUAACUCUGCAAU AUUAAA |
| C-Tag | Literature-guided: Direct Cbl binding; Crystal Structure (PDB ID 4FRN)[10] | GGACAUCGGUUUUAGUGGGGAACAGCCACUAAAAUAAUGGGGAAA GUUUGGUGCAAGUCCAACACUGUCCCGCAGCUGUAAGCAGACUAU CUCUGUGAGUCAGAACGCCCACCGAUGUCCCCCGUAAACACUUCU GCGAGGUACAGAAA |
| D-Tag | Literature-guided: Direct Cbl binding; Crystal Structure (PDB ID 4FRN)[10] | UACUGAAAGCGUGGUGGGAAACAAUGUGAAAGUCAUUGACUGUUC CUGCAACGGUAAGCGCUUCGGCGCGAGUCCGAAUGCCACCCAGUA AAGUCCGCUGUCGAGUGAAGGCCAGGAAAAGUCUAACUCA |
| E-Tag | Literature-guided: Direct Cbl binding; Vitamin B-12 biosynthesis[11] | UCCCCGUUAUACUGCUGUGGACAACUCGCCCGUGGUGCAGGAAGU CGGUUCAAGCCGACACGGUCGCGCCACUGUGAACAUUUCGGAGCA AAUGCCCCGCGAUGAGAGUCAGGAACUGGCCACGGACGAGCCUUU CAAGCGCGGGACGCACGAUCCCGAGAAAGGAAUGCCGGAAUGCCG UGCAUAUCGCA |
| F-Tag | Literature-guided: Indirect Cbl binding[1] | GAUUUCGGAACUAAAAAGAGGUUUAGUCAAUGAGGGAACGCUGUG CAAAUCGGCGACAGUACCCGCUGCUGUAAUUCUCUGUGAAUCCGC ACACUAUGUCACUGCAUCGGCAGAUGUGGGAAGGCGCUGCGGGGG AGGGAUAAGUCAGAAGACCUGCCGGAGUCAAAUAAACAUAUUAUU AAUCACUUUCGGGAGUUAAAGUU |
| G-Tag | Literature-guided: Indirect Cbl binding[1] | CCACUAACAUAAUUUGAUAGUGUAAGGGAAUGGGUGCUUUGUGCU UAAUAGGGAAGUCCGGUGUAAUACCGGCACGGUCCCGCCACUGUA AUGGGGAUGCUUCCUAAAUUUGCCACUGGGAAACCGGGAAGGCUU AGGGCGGCGAUGAACCAGAGUCAGGAGACCUGCCUGUUCUAACAC ACGCCGCUAGGACCUACGGAAGAUAGGCAGGUGUGGGUACAAUAA UCAGGCAAAAUGCAUAUAUUGUUUGGUGUUAUACCCCCUGCUAAG GGGGUUUUUCUA |
| H-Tag | Literature-guided: Indirect Cbl binding[1] | CUCAUGUCGCAAAACUACAUAUCGUGCAAAAAAGGUGCGCAAUGU UUACAUUGCGUUAAAUGGGAAGUCUGGUGUGAAGCCAGCGCGGUA CCCGCCACUGUGAAGAUGAGCUGAUUGCAUAUGCCACUGUAUUCG GGAAGGCGCAGUCAAGUGAUGAAUCGAAGUCAGGAAACCAGCCUU UUUUAAUGCGAAGCUUACCCUACGGGAUAUAGGUGGAAGUGCAAA AACUUAGUUUGAAUUUUUCUGCGCCUCUAUCAUUGAUAGAGGCGC UUUUUUUAGUAAAGGAGCGAAUGAGU |
| I-Tag | Literature-guided: Indirect Cbl binding[1] | AAGAUAAGACAAAUACAUAGCGUAAAGGUACAGGUGCCCGCAAGG GCUUCAUAGAAAAGCCGGUGAAAGGCCGGCGCGGUCCCGCCACUG UAAUGGGGAGCAAAGCCAAGAGAUGCCACUGGGAUACAAAUCCUG GGAAGGUUUGGCAGCGUAAUGAUCCAGAGCCAGGAGAACUGCCUG UACAACAAUCACCGUUGACCUGCGAAGAUGGGGAGGGGAUUUAAU |

| | | |
|---|---|---|
| | | GAACAAACAGCAGUAACCCCCCUAUGUCAUACAGGGGGGUUAUUUUAUUUACCGCUUGUCACCAUGUGGGACAAGCAAUGACUUACCAACAA |
| J-Tag | Literature-guided: Indirect Cbl binding[1] | UAAUAUUAUAGGUUCUUUAAGCAAGAUUAAUAGGGAAAAAGGUUAAAUUCCUUUACAGCCCCCGCUACUGUGAUGCAGACGAAACUUUUGUUAGCCACUAUGAUGAUAUUUUUUAUAUCUCAUGGGAAGGAAAAGGAGUAGGAUGAAGCUAAGUCAGGAGACCUGCCUAAAAUAUUAAAGUAAUUUCUUCGGGGAUGGAGAAAGUUUCUUAUAAUAAUUAUACAGAUAUGUUUGUUUUAUGUGUUUGUAUUUAGAUUGUGGAAAAUUAGAAGCCUAACCAUCGAGUUAGGCUUUUUUAUUUUAUUAAAUAAAUCAGACAUGUAUUAUUAUGUUUUAAUUAUUAGAAAGGGAGAGAGGGAUACUAAG |
| K-Tag | Literature-guided: Indirect Cbl binding[1] | AAAUAAUAAUAAAUUGCAUAAAGCAAGGAUAGGUGCCUACACAGGCAUAAUAGAGAAGACCGGUGCAAUACCGGCGCGGUCACGCCACUGUAAUGGGGAGCGAACUCAAAAAAUACCACUGGAAUGAAUGUUCUGGGAAGGUUUGAGUAAGCAAAGAGCCAAAGCCAGGAGAACUGCCUAUCUGAUAAUCACCGUUUUGACCUACGAGCGAUAGGAAGGGGAUUGACAUGUGUUUUUGUUAUAUUCCCCGGCUUGCAACCCCAAGUCGGCUUUUCUUUAUCCACGCAUGCCAAGUUGUCGGUCAGCUUUAACGGACCGGCAACUUUUUUUGUUCUUUUUGUAUACUUGGCAUACAAAAACACAUCAGACCAAUUAUUUUAAAGGAGGCAUAAAC |
| Atmut-Tag | Rationally designed: truncation at 3'-end; point mutations A67U A68U G70U | GGCCUAAAAGCGUAGUGGGAAAGUGACGUGAAAUUCGUCCAGAUUACUUGAUACGGUUAUACUCCGUUUUCCACCUAGGCC |
| At-Tag | Rationally designed; truncation at 3'-end | GGCCUAAAAGCGUAGUGGGAAAGUGACGUGAAAUUCGUCCAGAUUACUUGAUACGGUUAUACUCCGAAUGCCACCUAGGCC |
| Bt-Tag | Rationally designed; truncation at 3'-end | GGUACUGAAAGCGUGGUGGGAAACAAUGUGAAAUUCAUUGACUGUUCCUGCAACGGUAAAAGUAAAAUUGAGUCCGAAUGCCACCCAGUAAA |
| Ct-Tag | Rationally designed; truncation at 3'-end | GGACAUCGGUUUUAGUGGGGAACAGCCACUAAAAUAAUGGGGAAAGUUUGGUGCAAGUCCAACACUGUCCCGCAGCUGUAAGCAGACUAUCUCUGUGAGUCAGAACGCCCACCGAUGUCC |
| Dt-Tag | Rationally designed; truncation at 3'-end | GGUACUGAAAGCGUGGUGGGAAACAAUGUGAAAGUCAUUGACUGUUCCUGCAACGGUAAGCGCUUCGGCGCGAGUCCGAAUGCCACCCAGUAAA |
| N-Tag | Rationally designed; insertion mutation | GGCCGGCCUAAAAGCGUAGUGGGGGAAAGGUGACGCGUGAAAUUCGUCCAGAUUACUUGAUACGGCGGUUAUACUCCGCCGAAUGCCCCACCCCUAGGCCGGCCAUACAACGGAGGCAAGGAGACCUCC |
| S-Tag | Literature-guided: direct Cbl binding; SELEX experiment[12] | GGAACACUAUCCGACUGGCACCGCCAGCGGACAAAUCCGGUGCGCAUAACCACCUCAGUGCGAGCAACGAUGGCC |
| R-Tag | Rationally designed; point mutation U69C | GGCCUAAACGCAAAGUGGGAAAGUGACGUGAAAUUCGUCCAGAUUACUUGAUACGGUUAUACUCCGAAUGCCACCUAGGCCAUACAACAUACAACAUACAGAGCAAGGAGACUC |

| | | |
|---|---|---|
| Z-Tag | Rationally designed; point mutations A9C G12A U13A | GGCCUAAAAGCGUAGUGGGAAAGUGACGUGAAAUUCGUCCAGAUUACUUGAUACGGUUAUACUCCGAACGCCACCUAGGCCAUACAACAUACAACAUACAGAGCAAGGAGACUC |
| B12-Tag | Literature-guided: direct Cbl binding; crystal structure (PDB ID 1ET4)[13] | GGAACCGGUGCGCAUAACCACCUCAGUGCGAGCAA |

**Supplementary Table 2:** Primers used in PCR for RNA tag amplifications.

| Name | Forward Primer (5'→3') | Reverse Primer (5'→3') |
|---|---|---|
| A-Tag | GGATCCTAATACGACTCACTATAG | GAATTCGAGTCTCCTTGCT |
| B-Tag | GGATCCTAATACGACTCACTATAG | GAATTCTTTAATATTGCAGAGTTAGACTTT |
| C-Tag | GGATCCTAATACGACTCACTATAG | GAATTCTTTCTGTACCTCGCA |
| D-Tag | GGATCCTAATACGACTCACTATAG | GAATTCTGAGTTAGACTTTTCCTG |
| E-Tag | GGATCCTAATACGACTCACTATAG | GAATTCTGCGATATGCACG |
| F-Tag | GGATCCTAATACGACTCACTATAG | GAATTCAACTTTAACTCCCGAAAG |
| G-Tag | GGATCCTAATACGACTCACTATAG | GAATTCTAGAAAAACCCCCTTAGC |
| H-Tag | GGATCCTAATACGACTCACTATAG | GAATTCACTCATTCGCTCCTTT |
| I-Tag | GGATCCTAATACGACTCACTATAG | GAATTCTTGTTGGTAAGTCATTGC |
| J-Tag | GGATCCTAATACGACTCACTATAG | GAATTCCTTAGTATCCCTCTCTCC |
| K-Tag | GGATCCTAATACGACTCACTATAG | GAATTCGTTTATGCCTCCTTTAAAAT |
| Atmut-Tag | GGATCCTAATACGACTCACTATAG | GAATTCGGCCTAGGTGGAA |
| At-Tag | GGATCCTAATACGACTCACTATAG | GAATTCGGCCTAGGTGGC |
| Bt-Tag | GGATCCTAATACGACTCACTATAG | GAATTCTTTACTGGGTGGC |
| Ct-Tag | GGATCCTAATACGACTCACTATAG | GAATTCGGACATCGGTGGG |
| Dt-Tag | GGATCCTAATACGACTCACTATAG | GAATTCTTTACTGGGTGGCATTC |
| N-Tag | GGATCCTAATACGACTCACTATAG | GAATTCGGAGGTCTCCTTGC |
| S-Tag | GGATCCTAATACGACTCACTATAG | GAATTCGGCCATCGTTGC |
| R-Tag | GGATCCTAATACGACTCACTATAG | GAATTCGAGTCTCCTTGC |
| Z-Tag | GGATCCTAATACGACTCACTATAG | GAATTCGAGTCTCCTTGC |
| B12-Tag | GGATCCTAATACGACTCACTATAG | GAATTCTTGCTCGCACTGAGG |

(1) Kennedy, K. J.; Widner, F. J.; Sokolovskaya, O. M.; Innocent, L. V.; Procknow, R. R.; Mok, K. C.; Taga, M. E. Cobalamin Riboswitches Are Broadly Sensitive to Corrinoid Cofactors to Enable an Efficient Gene Regulatory Strategy. *mBio 13* (5), e01121-22. https://doi.org/10.1128/mbio.01121-22.

(2) Braselmann, E.; Wierzba, A. J.; Polaski, J. T.; Chromiński, M.; Holmes, Z. E.; Hung, S.-T.; Batan, D.; Wheeler, J. R.; Parker, R.; Jimenez, R.; Gryko, D.; Batey, R. T.; Palmer, A. E. A Multicolor Riboswitch-Based Platform for Imaging of RNA in Live Mammalian Cells. *Nat. Chem. Biol.* **2018**, *14* (10), 964–971. https://doi.org/10.1038/s41589-018-0103-7.

(3) Sarfraz, N.; Moscoso, E.; Oertel, T.; Lee, H. J.; Ranjit, S.; Braselmann, E. Visualizing Orthogonal RNAs Simultaneously in Live Mammalian Cells by Fluorescence Lifetime Imaging Microscopy (FLIM). *Nat. Commun.* **2023**, *14* (1), 867. https://doi.org/10.1038/s41467-023-36531-y.

(4) Sarfraz, N.; Shafik, L. K.; Stickelman, Z. R.; Shankar, U.; Moscoso, E.; Braselmann, E. Evaluating Riboglow-FLIM Probes for RNA Sensing. *RSC Chem Biol* **2024**. https://doi.org/10.1039/D3CB00197K.

(5) Sarfraz, N.; Lee, H. J.; Rice, M. K.; Moscoso, E.; Shafik, L. K.; Glasgow, E.; Ranjit, S.; Lambeck, B. J.; Braselmann, E. Establishing Riboglow-FLIM to Visualize Noncoding RNAs inside Live Zebrafish Embryos. *Biophys. Rep.* **2023**, *3* (4), 100132. https://doi.org/10.1016/j.bpr.2023.100132.

(6) Andronescu, M.; Condon, A.; Hoos, H. H.; Mathews, D. H.; Murphy, K. P. Efficient Parameter Estimation for RNA Secondary Structure Prediction. *Bioinformatics* **2007**, *23* (13), i19–i28. https://doi.org/10.1093/bioinformatics/btm223.

(7) Lu, Z. J.; Gloor, J. W.; Mathews, D. H. Improved RNA Secondary Structure Prediction by Maximizing Expected Pair Accuracy. *RNA* **2009**, *15* (10), 1805–1813. https://doi.org/10.1261/rna.1643609.

(8) Bellaousov, S.; Reuter, J. S.; Seetin, M. G.; Mathews, D. H. RNAstructure: Web Servers for RNA Secondary Structure Prediction and Analysis. *Nucleic Acids Res.* **2013**, *41* (W1), W471–W474. https://doi.org/10.1093/nar/gkt290.

(9) Reuter, J. S.; Mathews, D. H. RNAstructure: Software for RNA Secondary Structure Prediction and Analysis. *BMC Bioinformatics* **2010**, *11* (1), 129. https://doi.org/10.1186/1471-2105-11-129.

(10) Johnson Jr, J. E.; Reyes, F. E.; Polaski, J. T.; Batey, R. T. B12 Cofactors Directly Stabilize an mRNA Regulatory Switch. *Nature* **2012**, *492* (7427), 133–137. https://doi.org/10.1038/nature11607.

(11) Li, J.; Ge, Y.; Zadeh, M.; Curtiss, R.; Mohamadzadeh, M. Regulating Vitamin B12 Biosynthesis via the cbiMCbl Riboswitch in Propionibacterium Strain UF1. *Proc. Natl. Acad. Sci. U. S. A.* **2020**, *117* (1), 602–609. https://doi.org/10.1073/pnas.1916576116.

(12) Lorsch, J. R.; Szostak, J. W. In Vitro Selection of RNA Aptamers Specific for Cyanocobalamin. *Biochemistry* **1994**, *33* (4), 973–982. https://doi.org/10.1021/bi00170a016.

(13) Sussman, D.; Wilson, C. A Water Channel in the Core of the Vitamin B12 RNA Aptamer. *Structure* **2000**, *8* (7), 719–727. https://doi.org/10.1016/S0969-2126(00)00159-3.
